# Increasing the uptake of exercise programs in the dialysis unit: a protocol for a realist synthesis

**DOI:** 10.1186/s13643-016-0224-6

**Published:** 2016-04-21

**Authors:** Stephanie Thompson, Alex Clark, Anita Molzahn, Scott Klarenbach, Marcello Tonelli

**Affiliations:** University of Alberta, 3064-8308 114 Street, Edmonton, Alberta T6G 2V2 Canada; Faculty of Nursing, Level 3, Edmonton Clinic Health Academy, University of Alberta, 11405-87 Avenue, Edmonton, Alberta T6G 1C9 Canada; Division of Nephrology, University of Alberta, 11-107 CSB, Edmonton, Alberta T6G 2C3 Canada; Division of Nephrology, University of Calgary, TRW Building, 7th Floor, 3280 Hospital Drive NW, 7D12, Calgary, Alberta T2N 4Z6 Canada

**Keywords:** Realist synthesis, Contextual factors, Intradialytic exercise, Hemodialysis, Exercise

## Abstract

**Background:**

For people with end-stage kidney disease on hemodialysis, exercise during the dialysis treatment (intradialytic exercise) may promote exercise adherence and enhance aspects of the dialysis treatment. However, intradialytic exercise programs are complex and how to adapt program components to local context so that the program is more likely to attain its intended health outcomes have not been well described. To increase the uptake of exercise in clinical practice, more evidence is needed on how contextual factors influence the program’s impact.

**Methods:**

Using the realist approach, we aim to understand how the processes and structures of intradialytic exercise programs work to influence patient participation according to different contextual factors. The focus of a realist review is explanatory and aims to develop and test theory on how contextual factors trigger specific processes or behaviors (or “mechanisms”) to produce outcomes. Using the realist context-mechanism-outcome configuration of theory development, we will use a range of sources to develop initial candidate theories: a scoping review of published papers and the gray literature, and discussion with stakeholders. To provide a theoretical basis for how contextual factors could work to influence patient participation in intradialytic exercise (IDE), several of our preliminary theories will be based on dominant theories of exercise adherence and behavior change. To support or refute these initial theories, we will synthesize data from a systematic literature review and semi-structured interviews with intradialytic exercise program stakeholders, sampled from a range of programs worldwide.

**Discussion:**

The complexity of intradialytic exercise programs poses challenges to their implementation. Using the “context, mechanism, outcome” approach, the knowledge gained from this study will be used to develop general recommendations for renal care providers and administration on how to adapt components of an intradialytic exercise programs according to different contextual factors in order to promote patient participation.

**Systematic review registration:**

PROSPERO CRD42016033335

**Electronic supplementary material:**

The online version of this article (doi:10.1186/s13643-016-0224-6) contains supplementary material, which is available to authorized users.

## Background

Chronic kidney disease (CKD) is associated with high cardiovascular mortality [1] and markedly reduced quality of life [2]. Systematic reviews of interventions using regular exercise training suggest that exercise is a promising means of improving these outcomes in people with CKD: regular exercise is associated with improvements in cardiovascular fitness [3–5], heart-rate variability [4], and the physical dimension of quality of life (QoL) [3].

Despite these benefits, people with CKD report a low level of physical activity [6]. For those people with advanced kidney disease requiring dialysis, self-reported physical activity is below the fifth percentile of the general population [7]. One of the barriers to exercise participation in this population is the hemodialysis treatment itself, which necessitates 12–18 h per week spent in a health facility [8]. One means of effectively addressing this barrier is to recommend exercise during the dialysis treatment (intradialytic exercise (IDE)). Aside from convenience, there may be important advantages to performing exercise during the dialysis treatment, such as decreased severity of restless legs [9], improved dialysis adequacy [10], and increased enjoyment of dialysis time [11]. In clinical practice, however, IDE programs remain the exception rather than the rule. Several proposed explanations for the low uptake of IDE programs in clinical practice are as follows: the unknown effects of exercise on “hard” outcomes, such as survival [12]; the uncertainty on what exercise to recommend to patients for optimal benefit; and the methodological limitations of existing exercise randomized controlled trial (RCTs) specifically, the lack of blinded outcome assessment [3]. Yet even if these questions on efficacy are addressed, there is still a largely unaddressed evidence-practice gap about how to adapt the components of IDE programs to different contexts so that the program achieves its goals. This question is relevant for IDE program development because these programs have varying components, are heterogeneously delivered, and are implemented in complex and diverse settings—so what works in one setting may not work in another. A better understanding of the processes and structures that are necessary for the program to attain its effects can inform site-specific adaptation and also potentially enhance program effectiveness [13].

Although there is no absolute definition of what makes an intervention complex, the following description offers some guidance: complexity is introduced in an intervention by the number of interacting components; the number and difficulty of behaviors required by those delivering or receiving the intervention; the number of groups or organizational levels targeted by the intervention; the number and variability of outcomes; and the degree of adaptation or tailoring of the intervention to local context that occurs [14]. IDE programs satisfy this description of complexity. First, in addition to the exercises, exercise programs also include educational and psychological components. Second, IDE programs require dialysis unit staff to accommodate exercise (or exercise recommendations) into their workflow and for patients to exercise during a time that was previously restricted to sedentary activities. Third, to accommodate exercise in the dialysis unit, renal program managers may need to implement new unit policies and procedures. Fourth, the resources available for IDE programs will vary across different settings and will naturally result in local program adaptation. For example, there may be differences in the types of equipment that are available and in the skills and the experience of the staff who deliver the program (exercise therapists versus unit staff versus self-directed).

Understanding the complexity and context-sensitive nature of IDE programs has important implications for how these programs are evaluated. Although an RCT is the optimal study design to answer the question of whether the intervention works, it is not designed to answer the question of how a program achieves its effects. In addition, a potential barrier to the uptake of positive findings from RCTs is the insufficient information on the intervention and context [15]. To our knowledge, there have been no reviews in which the complex and multifaceted aspects of an IDE program or how these might work to influence program effectiveness have been systematically evaluated. This realist review aims to define the causal links between IDE programs and their intended outcomes (the program theory) and provide an understanding of what components of an IDE program are important for its success (or failure).

## Methods

The overall aim of this realist review is to understand how contextual factors trigger the mechanisms that influence patient participation in IDE programs. Mechanisms are the processes or structures that work according to specific contextual factors to generate an outcome of interest [16]. As different clinical IDE programs will use different outcomes to measure program effectiveness, the outcome of interest in this review is patient participation (recognizing that patient participation in IDE is necessary in order to obtain health benefits).

Our specific objectives are

- To identify the program theories on how IDE works to promote patient participation in exercise
- To identify the contextual factor(s) that triggered the mechanism(s) to influence patient participation in the IDE program
- To identify and explain the mechanisms that influence patient participation in IDE programs
- To use empiric data synthesized from a systematic review of the literature on IDE programs and interviews with IDE program stakeholders to test and refine our initial program theories

In addition, as patient participation in an IDE program represents only one stage of the implementation process, theories will also include contextual information on program development and delivery.

In general, programs are implemented with assumptions as to how they work to bring about their intended outcome(s). Realist review uses a systematic and theory-driven approach to refine these assumptions into theories that can then be empirically tested [17]. In a realist synthesis, the theory of how a program “works” is structured according to the “context-mechanism-outcome” (C-M-O) approach [18]. That is, the program theory is explained as the contextual (C) factor hypothesized to have triggered the relevant mechanisms (the underlying process or behavior) (M), to generate the outcome of interest (O) [19]. The process of a realist review is focused on identifying, explaining, and testing these semi-predictable C-M-O patterns (called demi-regularities) [17]. C-M-O configurations form the basis of the program theory known as “middle range” [20] theory. A middle range theory is abstract enough to be generalizable, but is also close enough to the data that it can be empirically tested. For example, a theory might emerge that HD units that have a dedicated exercise expert delivering IDE (the context) increase participants’ confidence in their physical capabilities and body knowledge (the mechanism), thereby facilitating regular participation in exercise (the outcome).

### Study design

**Fig. 1:**
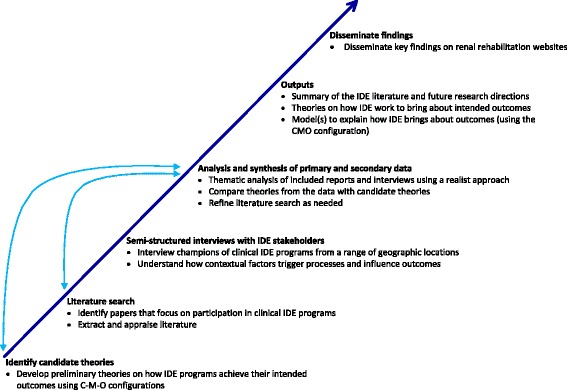
Overview of the stages of the realist review. The *arrows* indicate the iterative process of data collection, analysis, and theory development. *C-M-O* context-mechanism-outcome configuration

This realist review was based on the approach of Pawson et al. [17] and is consistent with publication standards for realist reviews (RAMESES criteria) [19]. An overview of the stages of the review is shown in Fig. 1.

This protocol has been written according to the PRISMA-P statement which is included as Additional file 1: PRISMA-P statement.

### Step 1: identify candidate theories

We will start with provisional program theories on how IDE works to facilitate patient participation in exercise. Development of these initial “candidate theories” [17] is a speculative process and will be refined in future stages of the review. We will use a number of methods and sources to derive these candidate theories: a rapid review of studies and reports on clinical IDE programs, examination of theories of behavior change related to exercise programs, and consultation with individual experts in the field. We will focus on identifying potentially influential contextual factors as defined by Pawson et al. [17], specifically, the individuals (skills, knowledge, and roles of those delivering the program); the lines of communication (among staff and different organizational levels); the institution (job descriptions or policies that incorporate aspects of the program); and the program’s resources (funding, space, equipment, and incentives).

### Step 2: literature search and data collection

To test our candidate theories, both primary and secondary data will be used. The literature review will include a broad range of sources and primary data will be collected through individual interviews with IDE stakeholders. The ethics review board at the University of Alberta has approved the study (Pro00057423).

### Literature search

The search strategy will include a systematic search of the literature. In searching the literature, we aim to identify papers that focus on patient participation in clinical IDE programs. We will use the skills of a specialist librarian to develop initial search terms, revise the search as required, and to identify relevant data sources. There will be no restrictions on publication type. The search will be restricted to the English language and will include a focused search of the unpublished and gray literature (i.e., thesis dissertations and renal rehabilitation websites). We will use hand-searching and pursue references of references. Studies that discuss participation in IDE programs only in terms of trial enrollment will not be included. ST will screen articles for inclusion based on title, abstract, and keywords against inclusion criteria. Potentially eligible studies will be obtained in full text and rescreened. A random subset of the full articles will be reviewed by another investigator. The decision to eliminate an article will be discussed with the study team, and reasons for exclusion will be documented.

### Semi-structured interviews

Our initial search of the literature indicates that few existing publications on IDE include sufficient information to inform the development of our initial program theories. Therefore, we will obtain additional information through individual, semi-structured interviews with IDE stakeholders. Using the results from our literature search, maximum variation sampling according to geographic location will be used to capture the variation in IDE programs. Where possible, we will also select sites based on known variation in contextual factors, such as program delivery and resources. We aim to interview champions of clinical IDE programs—those who play a key role in sustaining the programs. To identify these champions, we will contact the corresponding authors of the included studies or reports from our literature review. In cases where there is an individual perceived as more knowledgeable on the clinical aspects of the program, we will approach this individual for an interview. Eight to ten interviews are planned (or until theoretical saturation is reached). Potential participants will be informed about the study through an emailed letter of information and online access to the protocol. The decision to participate in the study implies consent. The interview questions will be piloted at two IDE programs and revised accordingly. All interviews will be transcribed verbatim for further analysis. The interview questions are aimed at understanding service provider perspectives on the link between processes, behaviors, and outcomes according to various contextual factors. To include IDE programs that are not represented in the literature, we will use snowball sampling. Interviewees will be asked for the names and contact information of sustainers in other IDE programs. The study will be advertised on select renal websites.

### Step 3: data extraction and study appraisal

The following manuscript characteristics will be extracted and tabulated on an excel spreadsheet: objectives, study design or publication type, size, setting, contextual components, mechanisms (how the intervention may have “worked” to trigger change), and manuscript quality. One author will extract data and another will check for accuracy. We are focusing on patient participation as an indicator of program effectiveness; however, we recognize programs may use different measures of effectiveness and we will discuss these outcomes in the analysis. Study quality will be judged according to quality standards appropriate for the type of research (rigor) and on whether the manuscript contributed to theory building (relevance) [17]. Two reviewers will evaluate the relevance and rigor of the included studies. Any disagreement will be resolved through consensus-based group discussions with the study team.

### Step 4: analysis and synthesis

A thematic approach will be used to identify patterns in context, mechanisms, and outcomes first within each document and then across documents. To identify demi-regularities, attention will be given to similarities and differences in outcomes across different contextual factors. Specifically, we aim to identify those demi-regularities that might act as barriers or enablers to IDE participation. Through discussion with the research team, we will identify the mechanisms by which these outcomes occur. We will test the demi-regularities to see if they are able to confirm, refute, or refine our candidate theories. Other approaches to test and refine our theories include comparisons with published evaluations from other disciplines that have incorporated exercise into routine care, such as cardiology and pulmonary medicine. If the data does not fully explain candidate theories or new theories emerge from the data, we will develop these theories further by refocusing the literature search.

## Discussion

This realist synthesis aims to explain how contextual factors influence the mechanisms of IDE programs to effect patient participation. Previous reports of IDE interventions have primarily focused on the exercise itself and not considered how the more complex and variable aspects of an IDE program might influence its impact. To our knowledge, this will be the first report that explicitly defines intradialytic exercise as a complex intervention. This distinction is important and has implications for research utilization. Focusing on IDE mechanisms shifts the focus away from the problematic goal of delivering a complex intervention in a standardized way to understanding how the components of an IDE program function to bring about their outcomes and which of these mechanisms are important to reproduce [13, 17]. How contextual factors modify the relation between mechanisms and outcomes is an additional advantage of the realist approach, as research users can understand how local resources may influence program impact.

One of the outputs from this review is a working model(s) that will explain the conditions for IDE program success. The model(s) will be constructed based on the C-M-O configuration, describing how different contextual factors work to influence the effectiveness of the IDE program [18]. All of the theories to describe how IDE programs work to increase patient participation will also be presented. Where possible, the theories generated from this review will be discussed alongside findings from systematic reviews on IDE and exercise chronic kidney disease.

The results of this review will be of interest to renal program administrators and a range of renal care providers. IDE programs are complex and potentially resource-intensive. Recommendations from this review on “what works and under what circumstances,” will provide useful information to administrators on where and how to allocate resources so that the program is more likely to attain its intended health outcomes. Although renal care providers acknowledge the importance of physical activity for patients with kidney disease, implementation of practices to increase uptake are low [21]. Generalizable lessons that can be used to address recognized barriers to patient participation in IDE e.g., lack of unit staff’s knowledge about IDE, difficulty motivating patients, and difficulty with existing resources, can improve the overall quality of IDE programs across a range of settings [21–23].

The outputs will be disseminated through a number of different mechanisms. To provide guidance on implementation of IDE programs, the key findings of this review will be posted on the websites of the following renal organizations: Canadian Association of Nephrology Nurses and Technologists (CANNT), Kidney Foundation of Canada, and various renal rehabilitation websites internationally.

## Additional file

Additional file 1: Recommended items to address in a systematic review protocol. PRISMA-P (Preferred Reporting Items for Systematic 355 review and Meta-Analysis Protocols) 2015 checklist. (DOC 88 kb)

## Abbreviations

CKD: chronic kidney disease
C-M-O: context-mechanism-outcome
IDE: intradialytic exercise
QoL: quality of life
RCT: randomized controlled trial
