# Supplementary material for: Increasing the uptake of exercise programs in the dialysis unit: a protocol for a realist synthesis
Source: Syst Rev. 2016 Apr 21;5:67. doi: 10.1186/s13643-016-0224-6 (PMC4839081; doi:10.1186/s13643-016-0224-6)
Supplement: Additional file 1: — Recommended items to address in a systematic review protocol. PRISMA-P (Preferred Reporting Items for Systematic 355 review and Meta-Analysis Protocols) 2015 checklist. (DOC 88 kb) [file 13643_2016_224_MOESM1_ESM.doc]

**PRISMA-P (Preferred Reporting Items for Systematic review and Meta-Analysis Protocols) 2015 checklist: recommended items to address in a systematic review protocol* Page numbers reference the clean version**

| Section and topic | Item No | Checklist item |
| --- | --- | --- |
| ADMINISTRATIVE INFORMATION | | |
| Title: |  |  |
| Identification | 1a | Identify the report as a protocol of a systematic review  **Increasing the uptake of exercise programs in the dialysis unit: a protocol for a realist synthesis** |
| Update | 1b | If the protocol is for an update of a previous systematic review, identify as such  **Not applicable** |
| Registration | 2 | If registered, provide the name of the registry (such as PROSPERO) and registration number  **CRD42016033335** |
| Authors: |  |  |
| Contact | 3a | Provide name, institutional affiliation, e-mail address of all protocol authors; provide physical mailing address of corresponding author  **Yes, please see title page** |
| Contributions | 3b | Describe contributions of protocol authors and identify the guarantor of the review  **“Authorship followed ICMJE guidelines. ST was responsible for the inception and design of the project and prepared the manuscript. AC, SK, AM and MT participated in the design of the study and provided methodological input. All authors read and approved the manuscript.” Page 13** |
| Amendments | 4 | If the protocol represents an amendment of a previously completed or published protocol, identify as such and list changes; otherwise, state plan for documenting important protocol amendments  **Changes will be made in the record on the PROSPERO website** |
| Support: |  |  |
| Sources | 5a | Indicate sources of financial or other support for the review |
| Sponsor | 5b | Provide name for the review funder and/or sponsor |
| Role of sponsor or funder | 5c | Describe roles of funder(s), sponsor(s), and/or institution(s), if any, in developing the protocol  **“a clinician fellowship award allocated to Dr. Stephanie Thompson from Alberta Innovates-Health Solutions funded this work. The funders had no role in the design, collection, analysis, interpretation, writing or submission of the manuscript.” Page 13** |
| INTRODUCTION | | |
| Rationale | 6 | Describe the rationale for the review in the context of what is already known  Please see Introduction, specifically pages 4 & 5: “**Yet even if these questions on efficacy are addressed, there is still a largely unaddressed evidence-practice gap about how to adapt the components of IDE programs to different contexts so that the program achieves its goals. This question is relevant for IDE program development because these programs have varying components, are heterogeneously delivered, and are implemented in complex and diverse settings – so what works in one setting may not work in another. A better understanding of the processes and structures that are necessary for the program to attain its effects can inform site-specific adaptation and also potentially enhance program effectiveness” pages 4 & 5** |
| Objectives | 7 | Provide an explicit statement of the question(s) the review will address with reference to participants, interventions, comparators, and outcomes (PICO).  **Page 7: “For example, a theory might emerge that HD units that have a dedicated exercise expert delivering IDE (the context) increase participants’ confidence in their physical capabilities and body knowledge (the mechanism) thereby facilitating regular participation in exercise (the outcome).”**  **For a realist review, the comparator is not relevant. The population is defined further on page 8.** **Please see first paragraph and objectives in the methods section Pages 6&7.** |
| METHODS | | |
| Eligibility criteria | 8 | Specify the study characteristics (such as PICO, study design, setting, time frame) and report characteristics (such as years considered, language, publication status) to be used as criteria for eligibility for the review  **Pages 8&9. Years considered is not stated as this will correspond to the years in the searched databases**. |
| Information sources | 9 | Describe all intended information sources (such as electronic databases, contact with study authors, trial registers or other grey literature sources) with planned dates of coverage  **Please see pages 8-10** |
| Search strategy | 10 | Present draft of search strategy to be used for at least one electronic database, including planned limits, such that it could be repeated  **Not available at this time** |
| Study records: |  |  |
| Data management | 11a | Describe the mechanism(s) that will be used to manage records and data throughout the review  **Mechanism to manage records is not provided.**  **“The following manuscript characteristics will be extracted and tabulated on an excel spreadsheet:” Page 10** |
| Selection process | 11b | State the process that will be used for selecting studies (such as two independent reviewers) through each phase of the review (that is, screening, eligibility and inclusion in meta-analysis)  **“ST will screen articles for inclusion based on title, abstract, and keywords against inclusion criteria. Potentially eligible studies will be obtained in full text and rescreened. A random subset of the full articles will be reviewed by another investigator. The decision to eliminate an article will be discussed with the study team and reasons for exclusion will be documented.” Page 9.** |
| Data collection process | 11c | Describe planned method of extracting data from reports (such as piloting forms, done independently, in duplicate), any processes for obtaining and confirming data from investigators  **“Our initial search of the literature indicates that few existing publications on IDE include sufficient information to inform the development of our initial program theories. Therefore, we will obtain additional information through individual, semi-structured interviews with IDE stakeholders**.” **Page 9** |
| Data items | 12 | List and define all variables for which data will be sought (such as PICO items, funding sources), any pre-planned data assumptions and simplifications  **“The following manuscript characteristics will be extracted and tabulated on an excel spreadsheet: objectives, study design or publication type, size, setting, contextual components, and mechanisms (how the intervention may have “worked” to trigger change) and manuscript quality. One author will extract data and another will check for accuracy. We are focusing on patient participation as an indicator of program effectiveness; however, we recognize programs may use different measures of effectiveness and we will discuss these outcomes in the analysis.” Page 10** |
| Outcomes and prioritization | 13 | List and define all outcomes for which data will be sought, including prioritization of main and additional outcomes, with rationale  **“We are focusing on patient participation as an indicator of program effectiveness; however, we recognize programs may use different measures of effectiveness and we will discuss these outcomes in the analysis.” Page 10** |
| Risk of bias in individual studies | 14 | Describe anticipated methods for assessing risk of bias of individual studies, including whether this will be done at the outcome or study level, or both; state how this information will be used in data synthesis  **“Study quality will be judged according to quality standards appropriate for the type of research (rigour) and on relevance (whether the manuscript contributed to theory building).(17) Two reviewers will evaluate the relevance and rigour of the included studies. Any disagreement will be resolved through consensus-based group discussions with the study team**.” **Page 10** |
| Data synthesis | 15a | Describe criteria under which study data will be quantitatively synthesised-**Not applicable for this type of review** |
|  | 15b | If data are appropriate for quantitative synthesis, describe planned summary measures, methods of handling data and methods of combining data from studies, including any planned exploration of consistency (such as I^2^, Kendall’s τ)**Not applicable for this type of review** |
|  | 15c | Describe any proposed additional analyses (such as sensitivity or subgroup analyses, meta-regression)**Not applicable for this type of review** |
|  | 15d | If quantitative synthesis is not appropriate, describe the type of summary planned  **“A thematic approach will be used to identify patterns in context, mechanisms, and outcomes first within each document and then across documents. To identify demi-regularities, attention will be given to similarities and differences in outcomes across different contextual factors. Specifically, we aim to identify those demi-regularities that might act as barriers or enablers to IDE participation. Through discussion with the research team, we will identify the mechanisms by which these outcomes occur. We will test the demi-regularities to see if they are able to confirm, refute, or refine our candidate theories. Other approaches to test and refine our theories include comparisons with published evaluations from other disciplines that have incorporated exercise into routine care, such as cardiology and pulmonary medicine. If the data does not fully explain candidate theories or new theories emerge from the data, we will develop these theories further by refocusing the literature search.” Page 11** |
| Meta-bias(es) | 16 | Specify any planned assessment of meta-bias(es) (such as publication bias across studies, selective reporting within studies) **Not applicable for this type of review** |
| Confidence in cumulative evidence | 17 | Describe how the strength of the body of evidence will be assessed (such as GRADE)  **GRADE system not relevant to this type of review. The quality of the evidence will be summarized as described in item 14.** |

*** It is strongly recommended that this checklist be read in conjunction with the PRISMA-P Explanation and Elaboration (cite when available) for important clarification on the items. Amendments to a review protocol should be tracked and dated. The copyright for PRISMA-P (including checklist) is held by the PRISMA-P Group and is distributed under a Creative Commons Attribution Licence 4.0.**

*From: Shamseer L, Moher D, Clarke M, Ghersi D, Liberati A, Petticrew M, Shekelle P, Stewart L, PRISMA-P Group. Preferred reporting items for systematic review and meta-analysis protocols (PRISMA-P) 2015: elaboration and explanation. BMJ. 2015 Jan 2;349(jan02 1):g7647.*
